# Endothelial Cells Promote Productive HIV Infection of Resting CD4^+^ T Cells by an Integrin-Mediated Cell Adhesion-Dependent Mechanism

**DOI:** 10.1089/aid.2021.0034

**Published:** 2022-02-04

**Authors:** Catherine M. Card, Bernard Abrenica, Lyle R. McKinnon, Terry Blake Ball, Ruey-Chyi Su

**Affiliations:** ^1^ JC Wilt Infectious Diseases Research Center, National Microbiology Laboratory, Public Health Agency of Canada, Winnipeg, Canada.; ^2^ Department of Medical Microbiology & Infectious Diseases, University of Manitoba, Winnipeg, Canada.; ^3^ Center for the AIDS Program of Research in South Africa (CAPRISA), Durban, South Africa.; ^4^ Department of Medical Microbiology, University of Nairobi, Nairobi, Kenya.

**Keywords:** integrin, LFA-1, VLA-4, endothelial cell, HIV susceptibility, CD4^+^ T cell

## Abstract

Resting CD4^+^ T cells are primary targets of early HIV infection events *in vivo*, but do not readily support HIV replication *in vitro*. This barrier to infection can be overcome by exposing resting CD4^+^ T cells to endothelial cells (ECs). ECs line blood vessels and direct T cell trafficking into inflamed tissues. Cell trafficking pathways have been shown to have overlapping roles in facilitating HIV replication, but their relevance to EC-mediated enhancement of HIV susceptibility in resting CD4^+^ T cells has not previously been examined. We characterized the phenotype of primary human resting CD4^+^ T cells that became productively infected with HIV when cocultured with primary human blood and lymphatic ECs. The infected CD4^+^ T cells were primarily central memory cells enriched for high expression of the integrins LFA-1 and VLA-4. ICAM-1 and VCAM-1, the cognate ligands for LFA-1 and VLA-4, respectively, were expressed by the ECs in the coculture. Blocking LFA-1 and VLA-4 on resting CD4^+^ T cells inhibited infection by 65.4%–96.9%, indicating that engagement of these integrins facilitates EC-mediated enhancement of productive HIV infection in resting CD4^+^ T cells. The demonstration that ECs influence cellular HIV susceptibility of resting memory CD4^+^ T cells through cell trafficking pathways engaged during the transmigration of T cells into tissues highlights the physiological relevance of these findings for HIV acquisition and opportunities for intervention.

## Introduction

The biological factors that favor HIV acquisition remain incompletely understood. Increased susceptibility to HIV infection has been linked with mucosal inflammation. Specifically, genital inflammation was a strong predictor of HIV acquisition in the CAPRISA 004 trial and inflammation undermined effectiveness of tenofovir gel in prevention of infection.^1,2^ Inflammation may promote HIV infection due to impairment of the mucosal barrier^3^ and recruitment of activated HIV target cells to mucosal sites.^4^ However, inflammation has additional effects on mucosal tissue and the resident stromal cells, including endothelial cells (ECs) and fibroblasts. These effects may also contribute to increased HIV susceptibility through the interactions between stromal cells and HIV target cells.

HIV preferentially replicates in activated CD4^+^ T cells, whereas resting CD4^+^ T cells demonstrate relative resistance to infection, particularly *in vitro*.^5–10^ However, nonhuman primate models have demonstrated that the initial founder population of SIV-infected cells is primarily resting CD4^+^ memory T cells.^11,12^ Furthermore, HIV latency is maintained in the resting memory CD4^+^ T cell population,^11–17^ although there is debate about whether this reservoir arises from infection of resting cells or return of infected activated cells to a resting state. These seemingly paradoxical findings may be reconciled by the observation that resting CD4^+^ T cells become susceptible to HIV infection *in vitro* when cultured in the presence of lymphoid tissue,^13^ implicating factors in the tissue microenvironment that can render resting cells more permissive to productive HIV infection. ECs, which are abundant in lymphoid and mucosal tissues, may partially account for this observation, as they have been shown to enhance productive and latent HIV infection of resting CD4^+^ T cells.^18–22^

There is no clear consensus yet on the mechanisms by which ECs enhance susceptibility of resting CD4^+^ T cells. Resistance of resting cells to infection results from a combination of restriction factors and low expression of HIV dependency factors.^23^ A previous study found EC-mediated enhancement of resting CD4^+^ T cell infection to be independent of changes to restriction factor expression.^21^ In early studies using an allogeneic stimulation model, expression of major histocompatibility complex class II (MHC-II) on ECs partially contributed to enhancement of infection,^18,19^ but resting ECs, which lack MHC-II expression, were also shown to enhance infection by cell contact-dependent and -independent mechanisms.^20,21^ Among soluble factors tested, interleukin (IL)-6 was implicated as a contributor to enhancement of infection, but other unidentified factors may also play a role under specific conditions.^21^

ECs are nonhematopoietic cells that line the vessels of the blood and lymphatic vascular systems. Although ECs play multiple roles in shaping immune responses, they are perhaps best known for their function as mediators of cell homing to tissues and lymph nodes. This occurs through production of chemokine gradients, which direct cellular migration and binding of lymphocyte integrins and selectins to their cognate endothelial-expressed cellular adhesion molecule (CAM) ligands. Such EC adhesion molecules include E-selectin, ICAM-1, VCAM-1, and MAdCAM-1. Under inflammatory conditions, cytokines, such as TNFα and IL-1β, produced by immune and stromal cells in tissue lead to changes in the endothelium, including upregulation of ICAM-1 and VCAM-1, thereby promoting recruitment of T cells bearing their cognate ligands, integrins LFA-1 (αLβ2) and VLA-4 (α4β1), respectively.

Cell homing pathways have previously been implicated in HIV susceptibility and pathogenesis. HIV preferentially targets CD4^+^ T cells expressing VLA-4 and the gut-homing integrin α_4_β_7_.^24–30^ Blocking of α_4_β_7_ in monkey models showed promise for prevention of SIV infection and for viral control in SIV-infected monkeys.^31,32^ However, the latter results were not substantiated in recent studies of nonhuman primates^33–35^ or HIV-infected patients.^36^ LFA-1 has also been implicated in HIV pathogenesis, and has been recognized as a mediator of virus attachment^37–39^ and cell/cell spread of HIV.^40–42^ While these data implicate integrins as markers of target cells in HIV infection, further investigation of the mechanisms by which they mediate HIV susceptibility is needed.

Given the relevance of cell homing pathways to HIV pathogenesis and the role of ECs in regulating cell trafficking, we hypothesized that molecules that mediate cell homing may also mediate the enhancement of HIV infection observed when resting CD4^+^ T cells are cultured with ECs. To test this hypothesis, we cultured resting CD4^+^ T cells in the presence of blood and lymphatic ECs, and subsequently infected them with HIV *in vitro*. We show here that resting CD4^+^ T cells expressing high levels of integrins LFA-1 and VLA-4 are preferentially infected. Targeted blocking of these integrins prevented adhesion of CD4^+^ T cells to ECs and diminished the EC-mediated enhancement of productive HIV infection.

## Materials and Methods

### Study subjects and ethics statement

Healthy blood donors were recruited locally in Winnipeg, Canada. Written consent was obtained from all study participants. The Research Ethics Board at the University of Manitoba approved the study protocols. For some experiments, whole blood was purchased from StemCell Technologies (Vancouver, Canada). The study protocols were approved by Research Ethics Boards at University of Manitoba (HS21429) and Health Canada/Public Health Agency of Canada (2020-014P).

### Cell isolation and culture

Peripheral blood mononuclear cells (PBMC) were isolated from EDTA-whole blood by density gradient centrifugation (*n* = 12) using Lymphoprep and SepMate tubes (StemCell Technologies). Resting CD4^+^ T cells (rCD4s) were isolated from PBMC by magnetic negative selection using the EasySep Human Resting CD4^+^ T cell Isolation Kit (StemCell Technologies). In some experiments, bulk CD4^+^ T cells were isolated using the EasySep CD4^+^ T cell Enrichment Kit (StemCell Technologies). Isolated CD4^+^ T cells were maintained in RPMI medium supplemented with 10% fetal bovine serum and 1% penicillin/streptomycin (Thermo Fisher, Burlington, Canada). Purity of isolated cells was assessed by flow cytometry.

Primary human umbilical vein endothelial cells (HUVEC) and human dermal microvascular lymphatic endothelial cells (LECs) were obtained from Lonza (Walkersville, MD) and cultured in Lonza EGM-2 or EGM-2MV complete culture media, respectively (Cedarlane, Burlington, Canada). ECs were seeded at 5,000 cells/cm^2^ and cultured to at least 70% confluence before coculture with CD4^+^ T cells.

### Viruses

The following viruses were originally obtained through the NIH AIDS Reagent Program, Division of AIDS, NIAID, NIH: HIV-1_IIIB_, ARP398, contributed by Dr. Robert Gallo, and HIV-1_BaL_, from Dr. Suzanne Gartner, Dr. Mikulas Popovic, and Dr. Robert Gallo.^43^ Viruses were propagated in PBMCs that had been stimulated for 3 days with 5 μg/mL PHA-L (Millipore Sigma, Oakville, Canada) and 20 U/mL IL-2 (Thermo Fisher). Virus stocks were harvested on day 8 (HIV_IIIB_) or day 11 (HIV_BaL_) postinfection. Titers of harvested stocks were performed by TCID50 using PHA-stimulated PBMC from a minimum of two separate donors.

### EC-rCD4 cocultures and infection assays

ECs were cultured to at least 70% confluence. Where indicated, ECs were treated with 100 U/mL TNFα (R&D Systems, Toronto, Canada) overnight to maximize expression of cell adhesion molecules before coculture. Media containing TNFα was then aspirated and the EC monolayers were rinsed with phosphate-buffered saline (PBS) to remove residual TNFα before coculture with rCD4s. At the time of coculture, 300,000 rCD4s were added per well to EC monolayers in a 48-well plate. Where indicated, rCD4 cells were preincubated with 10 μg/mL anti-αL clone TS1/22 (Thermo Fisher ) + 2.5 μg/mL anti-α4 clone 2B4 (R&D Systems) or 12.5 μg/mL mouse IgG1κ isotype control, clone 11711 (R&D Systems) for 30 min before being added to ECs. Blocking antibodies were maintained in the coculture throughout the experiment.

Cocultures were maintained in RPMI medium supplemented with 10% fetal bovine serum and 1% penicillin/streptomycin. After 24 h, cocultures were exposed to HIV_IIIB_ or HIV_BaL_ at a multiplicity of infection (MOI) of 1.0 for 4 h. To remove input virus, cocultures were centrifuged for 5 min at 400 × *g*, which is sufficient to pellet cells but leaves viral particles in suspension. Supernatant was discarded, then PBS was added, and the plates were centrifuged and supernatant discarded again. After removal of input virus, RPMI medium supplemented with 10% fetal bovine serum, 1% penicillin/streptomycin, and 10 U/mL recombinant IL-2 was added back to wells and cocultures were incubated for 3 days. Control cultures of rCD4 alone were treated identically to matched cocultures. On day 3 postinfection, rCD4s were collected and analyzed by flow cytometry. Supernatants were collected from culture wells, treated with 1% Triton X-100 (Millipore Sigma) and stored at −80°C.

In experiments evaluating transinfection, TNFα-treated ECs were exposed to an inoculum of HIV_IIIB_ equivalent to that dispensed in cocultured infections for 1 h at 37°C. Following incubation, the viral inoculum was aspirated then the EC monolayers were rinsed with PBS to remove input virus. rCD4 were then added to the EC monolayers and cocultured as above. Where indicated, rCD4 cells were preincubated with anti-αL and anti-α4 blocking antibodies or isotype control antibody for 30 min before being added to ECs, as above.

### Flow cytometry

Cells were washed, then incubated with a cocktail of cell surface antibodies at 4°C for 30 min. Cells were then washed and either fixed with 1% paraformaldehyde (Fisher Scientific, Ottawa, Canada) for 30 min or further stained for intracellular antigens. Intracellular staining of HIV-p24 and Ki67 was performed using the eBioscience Foxp3/Transcription Factor Staining Buffer set (Thermo Fisher ). A list of flow cytometry panels and antibodies used is provided in Supplementary Table S1. Samples were acquired on a BD LSRII using FACSDiva software (BD Biosciences, Mississauga, Canada) and data were analyzed using FlowJo software. Positive fluorescence signals were established using fluorescence minus one (FMO) controls. Visualization of coexpression analysis was performed using SPICE software.^44^

### HIV-p24 enzyme-linked immunosorbent assay

Culture supernatants were diluted 20-fold with PBS +1% BSA +0.2% Triton X-100 then analyzed for HIV-p24 protein using the HIV-1 Gag p24 DuoSet Enzyme-Linked Immunosorbent Assay (ELISA) Development System (R&D Systems) with the R&D TMB substrate reagent pack for detection, according to the manufacturer's instructions. Reaction was stopped using 2N H_2_SO_4_, then optical density at 450 nm with wavelength correction at 570 nm was acquired using a Synergy H1 plate reader (BioTek, Winooski, VT).

### Static adhesion assay

Titration of blocking antibodies was performed by using static adhesion assays, in which activated CD4^+^ T cells were allowed to adhere to either recombinant human ICAM/Fc or VCAM/Fc chimeras in the presence of blocking antibodies specific for integrin α4 (clone 2B4; R&D Systems) or integrin αL (clone TS1/22; Thermo Fisher) or mouse IgG1κ isotype control (clone 11711; R&D Systems). Wells were coated with 5 μg/mL of either rhICAM/Fc chimera (R&D Systems) or rhVCAM/Fc chimera (R&D Systems) overnight at 4°C. Plates were washed, then blocked with PBS containing 2% Goat Serum (Thermo Fisher ) and 0.01% Tween-20 (Millipore Sigma) for 2 h at 37°C. Purified CD4^+^ T cells were counted and labeled with 2 μM Cell Tracker Green CMFDA dye (Thermo Fisher) for 20 min at 37°C.

Cells were then stimulated overnight with 100 ng/mL PMA and 1 μM ionomycin (StemCell Technologies). Activated CD4^+^ T cells were preincubated with blocking antibodies at concentrations ranging from 10 to 1.25 μg/mL for 30 min at 37°C. Cells were transferred to rhCAM/Fc-coated plates (5 × 10^4^/well) and allowed to adhere in the presence or absence of blocking antibody or isotype control for 30 min at 37°C. Wells were gently mixed and supernatant was aspirated to remove nonadherent cells. Wells were washed gently twice with PBS then adherent cells were visualized using GFP channel on an EVOS fluorescent microscope (Thermo Fisher). Digital images were captured under the same magnification and intensification. Image processing was restricted to adjustment of brightness and contrast using Adobe Photoshop and all images acquired under the same conditions were treated identically. Cell-tracker green labeled cells were counted using Cell Profiler software.^45^ A minimum of 3 fields was counted per well in a 96-well plate.

To confirm effect of blocking in the EC-rCD4 coculture experiments, the static adhesion assay was performed using HUVEC monolayers instead of rhCAM/Fc chimeras. ECs were grown to at least 70% confluence then media were aspirated and wells were rinsed with PBS. Purified rCD4 were labeled with Cell Tracker Green CMFDA dye as above. Labeled rCD4 were preincubated with blocking antibodies (10 μg/mL anti-αL clone TS1/22 + 2.5 μg/mL anti-α4 clone 2B4) or isotype control antibody (12.5 μg/mL mouse IgG1κ clone 11711) for 30 min where indicated, then added to EC monolayers and allowed to adhere in the presence of antibody for 30 min at 37°C. As above, wells were mixed gently, then nonadhered cells were aspirated and wells were washed twice before visualization and image capture on an EVOS microscope.

### Statistical analyses

**FIG. 1.**
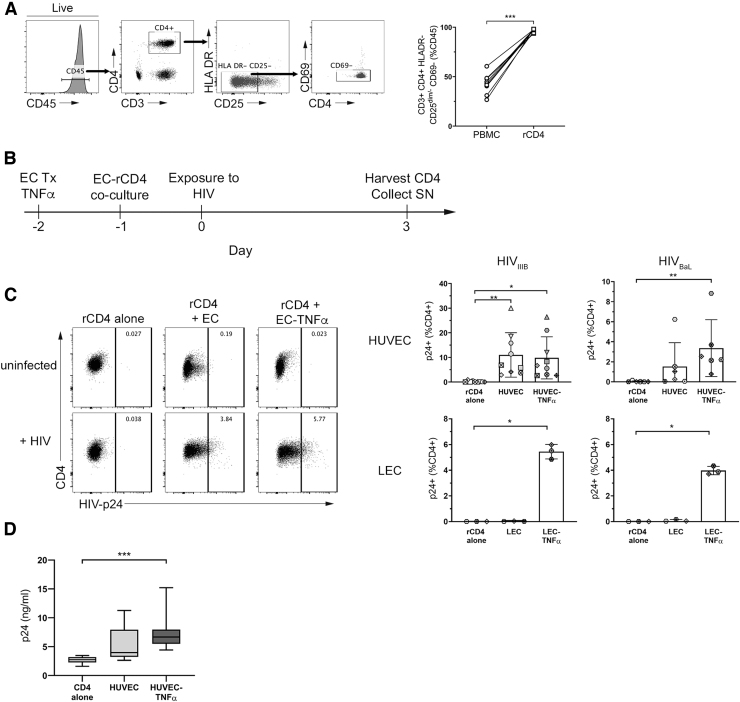
ECs promote HIV infection of rCD4. **(A)** Representative flow cytometry data showing gating of CD4^+^ HLA DR^−^ CD25dim/^−^ CD69^−^ T cells in PBMC. Gating for singlets, lymphocytes, and live CD45^+^ cells was performed before analyzing the indicated markers. Graph shows quantification of the purity of resting CD4^+^ T cells in the indicated sample types (*n* = 9). **(B)** Schematic timeline of EC-rCD4 coculture infection assay. **(C)** Representative flow cytometry data demonstrating intracellular detection of HIV-p24 protein in rCD4 cells cultured alone or in the presence of untreated or TNFα-treated HUVECs. Gating for singlets, lymphocytes, live cells, CD45^+^, and CD4^+^ was performed before analyzing HIV-p24. Graphs show proportions of rCD4 cells that were HIV-p24^+^ following HIV_IIIB_ or HIV_BaL_ infection in the presence of indicated ECs. Data points indicate different CD4^+^ T cell donors and each donor is indicated by a different symbol (*n* = 3–9). **(D)** Quantification of HIV-p24 protein in cell culture supernatants from HUVEC cocultures with HIV_IIIB_ infection by HIV-p24 ELISA (*n* = 9). Statistics assessed by Friedman **(B)** or Kruskal–Wallis **(C)** test and Dunn's post-test (*p < .05, **p < .01, ***p < .001 in post-test comparisons). ECs, endothelial cells; ELISA, enzyme-linked immunosorbent assay; HUVEC, human umbilical vein endothelial cell; PBMC, peripheral blood mononuclear cells; rCD4, resting CD4^+^ T cell.

Continuous variables in matched samples were analyzed using nonparametric paired Friedman test followed by Dunn's multiple comparisons test when analyzing three or more groups. For the analysis of ELISA data shown in Figure 1, nonparametric unpaired Kruskal–Wallis test had to be used due to lack of supernatant available for analysis of the HUVEC condition for two participants. For comparison of two matched samples, the nonparametric Wilcoxon matched-pairs signed-rank test was used. Differences were considered to be statistically significant if *p* < .05. All statistical analyses were performed using GraphPad Prism software, version 8, except for comparisons of phenotypes in the coexpression analyses, which were done within SPICE software.^44^ In those analyses, asterisks indicate statistically significant differences meeting the threshold for Bonferroni correction.

## Results

### ECs enhance infection of resting CD4^+^ T cells

Resting CD4^+^ T cells (rCD4) were isolated from PBMC from healthy donors (*n* = 12). Magnetic negative separation resulted in a rCD4 population that was CD3^+^ CD4^+^ HLA DR^−^ CD25^−^/dim CD69^−^ (median purity 97.2% of total CD45^+^ cells; Fig. 1A). rCD4 cells were further phenotyped to determine baseline expression of markers of memory (CD45RA, CCR7), activation (CD69, Ki67), integrins VLA-4 (β1), LFA-1 (β2) and α4β7 (β7), and CCR6 on rCD4 and compared with bulk CD4^+^ T cells in PBMC (Supplementary Table S2). Compared with bulk CD4^+^ T cells, a greater proportion of isolated rCD4 cells expressed CD45RA (*p* = .004). These CD45RA^+^ cells were almost universally CCR7^+^ in both PBMC and isolated rCD4 (medians 97.7% and 99.3%, respectively), indicating a naive phenotype. Compared with bulk PBMC, the proportion of CD45RA^+^ cells that expressed CCR7 was significantly higher in isolated rCD4 cells (*p* = .004). This was expected, as naive cells, by definition, which have not previously been activated by their cognate antigen are therefore expected to be quiescent. Within the CD45RA^+^ naive population, greater proportions of rCD4 cells also expressed integrin β7 (*p* = .043) compared with bulk CD4^+^ T cells in PBMC.

Among CD45RA^−^ memory cells, greater proportions of rCD4 cells were CCR7^+^ (*p* = .004), indicating a central memory phenotype. The CD45RA^−^ memory rCD4 cells were also enriched for integrin β1^hi^ (*p* = .027), integrin β7^hi^ (*p* = .004), and CCR6^+^ (*p* = .012) populations compared with bulk CD4^+^ T cells in PBMC. CCR6 expression density was higher on CCR6^+^ rCD4 than CCR6^+^ bulk CD4^+^ T cells [median fluorescence intensity (MFI); *p* = .012]. In addition, fewer isolated rCD4 expressed the early activation marker CD69 (*p* = .02) or the cell cycle marker Ki67 (*p* = .002) compared with bulk CD4^+^ T cells, consistent with depletion of activated cells from PBMC (Supplementary Table S2).

Isolated rCD4 cells were cocultured with resting or TNFα-treated ECs derived from blood (HUVEC) or lymphatic (LEC) vessels for 24 h before exposure of cocultures to HIV_IIIB_ or HIV_BaL_. Three days postinfection, cells were collected to assess frequencies and phenotypes of HIV-infected cells, identified by intracellular expression of the HIV core protein, Gag p24 (Fig. 1B). As expected, productively infected cells were not detectable when rCD4 cultured alone were exposed to HIV_IIIB_ or HIV_BaL_ (Fig. 1C). However, consistent with previous work,^18–22^ coculture with HUVEC or TNFα-treated HUVEC resulted in a significant increase in the proportion of cells productively infected with HIV_IIIB_ or HIV_BaL_ (*p* = .0002, *p* = .0017, respectively; Fig. 1C, top panel).

Coculture of rCD4 with TNFα-treated LEC similarly increased the proportion of cells productively infected with HIV_IIIB_ or HIV_BaL_ (*p* = .0278 for both, Fig. 1C, bottom panel). Notably, this only occurred when LECs were pretreated with TNFα, such that resting LECs did not promote productive infection. Consistent with these data, significantly more HIV-p24 protein was detectable in culture supernatants from rCD4 cells cocultured with TNFα-treated HUVEC compared with those cultured alone (*p* = .0003; Fig. 1D).

### High expression of integrins VLA-4 and LFA-1 marks HIV-susceptible cells in EC cocultures

To identify markers of susceptible HIV target cells, we further examined markers of memory (CD45RA, CCR7), activation (CD69, Ki67), integrins VLA-4 (β1), LFA-1 (β2) and α4β7 (β7), and CCR6 on rCD4 cells that became productively infected (HIV-p24^+^) and those that remained uninfected (HIV-p24^−^) following HIV_IIIB_ exposure in the presence of TNFα-treated HUVECs (gating strategy shown in Supplementary Fig. S1).

The majority of infected (HIV-p24^+^) cells had a central memory phenotype (CD45RA^−^ CCR7^+^; median 52.5%). This was in contrast to uninfected (HIV-p24^−^) cells, which were primarily CD45RA^+^ CCR7^+^ (87.2%), consistent with a naive phenotype (HIV-p24^+^ vs. HIV-p24^−^ *p* = .004; Table 1). Infected cells were enriched for high expression of integrins VLA-4 (as indicated by β1 expression), LFA-1 (β2) and β7, as well as CCR6 and the activation marker CD69 (*p* = .004; Table 1). Although cells expressing the proliferation marker Ki67 were also enriched in the infected cell population (*p* = .004; Table 1), they were relatively rare (range 1.71%–3.11% of HIV-p24^+^ cells), suggesting that the infected cells were not actively proliferating.

**Table 1.** Comparison of Phenotypes Between HIV-p24^−^ and HIV-p24^+^ CD4^+^ T Cells in Cocultures with TNFα-Endothelial Cell

Parameter^a^	HIV-p24^−^	HIV-p24^+^	*p* ^b^
CD45RA^−^ CCR7^+^ (%)	7.1 (4.4–10.3)	52.5 (38.1–71.0)	**.004**
CD45RA^+^ CCR7^+^ (%)	87.2 (81.4–92.3)	33.2 (19.9–47.3)	**.004**
β1^hi^ CD45RA^−^ (%)	5.0 (2.1–11.1)	43.3 (32.2–57.3)	**.004**
β1 MFI	2,709 (2,500–3,046)	3,460 (2,946–3,561)	**.004**
β1^lo^ CD45RA^−^ (%)	4.2 (2.6–6.6)	14.6 (11.6–18.5)	**.004**
β1 MFI	924.5 (895.0–945.0)	1,133 (1,091–1,163)	**.004**
β1^lo^ CD45RA^+^ (%)	88.4 (83.3–93.4)	32.8 (16.7–42.2)	**.004**
β1 MFI	833.0 (786.5–953.0)	988.0 (875.5–1,160)	**.004**
β2^hi^ CD45RA^−^ (%)	8.9 (4.6–12.4)	63.8 (49.1–77.0)	**.004**
β2 MFI	2,500 (2,223–2,709)	11,247 (4,008–14,093)	**.004**
β2^+^ CD45RA^+^ (%)	90.5 (85.8–94.3)	35.5 (16.4–44.5)	**.004**
β2 MFI	4,516 (220.0–5,241)	5,506 (1,882–6,399)	**.004**
β7^hi^ CD45RA^−^ (%)	2.6 (1.2–3.5)	16.3 (11.3–18.1)	**.004**
β7 MFI	4,220 (2,069–4,903)	4,319 (2,063–4,530)	.250
β7^int^ CD45RA^+^ (%)	53.6 (42.4–58.6)	22.3 (12.0–28.9)	**.004**
β7 MFI	914.0 (415.0–1,251)	1,070 (443.5–1,408)	**.020**
CCR6 (%)	1.07 (0.28–2.6)	34.3 (13.6–45.1)	**.004**
CCR6 MFI	1,312 (1,196–1,390)	2,099 (1,839–2,225)	**.004**
CD69 (%)	1.1 (0.42–2.0)	33.8 (22.9–37.0)	**.004**
Ki67 (%)	0.04 (0.02–0.06)	2.7 (2.0–3.0)	**.004**

^a^ Expression values listed as median of % positive (+) cells within HIV-p24^+^ or HIV-p24^−^ parent population or MFI of indicated cell population, with interquartile range shown in parentheses.

^b^ *p*-Values calculated using nonparametric Wilcoxon matched pairs test. Parameters considered statistically significant (*p* < .05) are highlighted in bold text.

MFI, median fluorescence intensity.

**FIG. 2.**
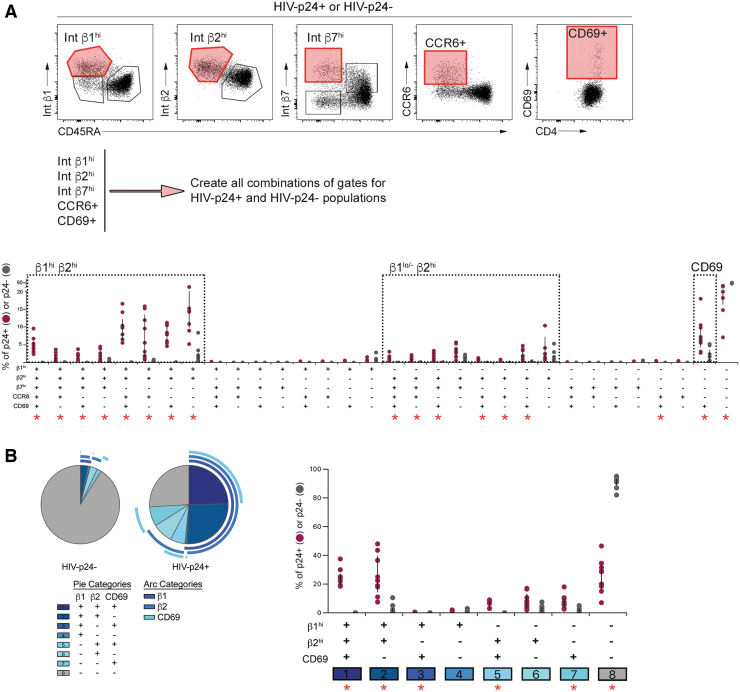
Coexpression analysis of phenotypic markers in HIV-infected and uninfected rCD4. **(A)** A Boolean gating strategy was applied to the populations with the highest expression of integrins β1, β2 and β7, CCR6, and CD69 (tinted *red* gates in flow cytometry plots, *top panel*) in both infected (HIV-p24^+^) and uninfected (HIV-p24^−^) subsets from rCD4 cells exposed to HIV_IIIB_ in the presence of TNFα-treated HUVECs. Gating for singlets, lymphocytes, live cells, CD45^+^, and CD4^+^ was performed before analyzing HIV-p24 and the indicated markers. Comparison of HIV-infected (HIV-p24^+^, *purple circles*) and uninfected (HIV-p24-, *gray circles*) cells for each phenotypic combination (*bottom panel*). **(B)** Coexpression analysis of integrins β1 and β2 and CD69 among HIV-infected and uninfected cells. Pie graphs indicate relative contribution of each combination to uninfected (HIV-p24^−^) or infected (HIV-p24^+^) population. Each marker is indicated by an arc surrounding the graph. Overlapping arcs indicate coexpression. Graph shows comparison of HIV-infected (HIV-p24^+^, *purple circles*) and uninfected (HIV-p24^−^, *gray circles*) cells for each phenotypic combination. Statistical comparisons were performed by Wilcoxon matched-pairs signed-rank test. *Red asterisks* indicate significant comparisons after Bonferroni correction [*p* < .002 for **(A)**, *p* < .006 for **(B)**].

We next evaluated whether these were exclusive markers of different HIV-susceptible subset cells or whether the same subset of HIV-susceptible cells coexpressed multiple enriched markers. To achieve this, a Boolean gating strategy was applied to the populations with the highest expression of each enriched marker in both infected (HIV-p24^+^) and uninfected (HIV-p24^−^) subsets from rCD4 cells exposed to HIV_IIIB_ in the presence of TNFα-treated HUVECs (Fig. 2A).

As Ki67 expression was low on both infected and uninfected cell subsets, it was excluded from coexpression analysis. The infected cell population was heterogenous and comprised numerous subsets defined by combinations of the specified markers (Fig. 2A). There was also a subset of cells that did not express high levels of any of the specified markers, which was highly enriched among cells that remained uninfected after HIV-exposure (median 28.6% of HIV-p24^+^ vs. 92.5% of HIV-p24^−^ cells; *p* = .004). Despite this heterogeneity, three main phenotypic groupings were evident based on expression of β_1_ and β_2_ integrins. Cell subsets that were β1^hi^ β2^hi^ and β1^lo/−^ β2^hi^ were enriched among infected compared with uninfected cells, and had variable expression of β7, CCR6, and CD69 (Fig. 2B). A third phenotype that was overrepresented among infected cells was marked solely by CD69 expression in the absence of high levels of β1 and β2 integrins (Fig. 2A).

These subsets were clearly represented when we repeated the coexpression analysis to focus specifically on β1, β2, and CD69 (Fig. 2B). Specifically, cells that were either β1^hi^ β2^hi^ CD69^+^ or β1^hi^ β2^hi^ CD69^−^ accounted for approximately half of infected cells, but fewer than 2% of their uninfected counterparts (HIV-p24^+^ vs. HIV-p24^−^ *p* = .0003, *p* = .0005 for CD69^+^ and CD69^−^ subsets, respectively; Fig. 2B). The infected cell population was further enriched for β1^lo/−^ β2^hi^ CD69^+^ cells and other minor subsets, including cells that expressed CD69 in the absence of high levels of β1 and β2 integrins (Fig. 2B).

**FIG. 3.**
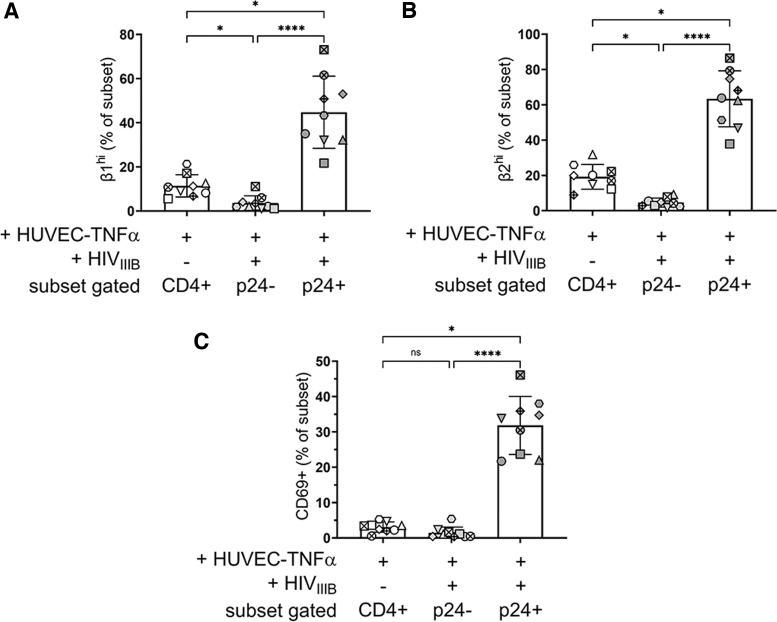
rCD4 expressing high levels of integrins VLA-4 and LFA-1 are preferentially targeted by HIV in the presence of ECs. Comparison of rCD4 cells expressing **(A)** high levels of VLA-4 (β1^hi^), **(B)** high levels of LFA-1 (β2^hi^) or **(C)** CD69 between rCD4 cells cocultured with TNFα-treated HUVEC but unexposed to HIV_IIIB_ and those that became infected (p24^+^) or remained uninfected (p24) following exposure to HIV_IIIB_. Statistics assessed by Friedman test and Dunn's post-test (*p < .05, ****p < .0001 in post-test comparisons).

To address whether CD4^+^ cells expressing high levels of β1 and β2 integrins and CD69 were preferentially targeted by HIV, or whether these proteins were upregulated in HIV-infected cells as a consequence of viral replication, we evaluated the proportions of β1^hi^, β2^hi^, and CD69^+^ cells among rCD4 cells that had been cocultured with TNFα-treated EC, but had not exposed to HIV, and compared these with the infected (HIV-p24^+^) and uninfected (HIV-p24^−^) rCD4 populations in cocultures exposed to HIV (Fig. 3).

Compared with rCD4 from cocultures not exposed to HIV, β1^hi^ and β2^hi^ cells were underrepresented in the HIV-p24^−^ subset (*p* = .004) and overrepresented in the HIV-p24^+^ subset (*p* = .004) of rCD4 cells from cocultures exposed to HIV (Fig. 3A, B, respectively), which may suggest preferential infection of these cells. In contrast, there was no significant difference in CD69 expression between rCD4 from cocultures not exposed to HIV and the HIV-p24^−^ subset of rCD4 cells from cocultures exposed to HIV (Fig. 3C). The HIV-p24^+^ subset was enriched for CD69^+^ cells, suggesting upregulation of CD69 following infection rather than preferential infection of CD69^+^ target cells.

Collectively, these data implicate β1^hi^ β2^hi^ and β1^lo/−^ β2^hi^ cells as the primary targets for HIV infection in the presence of ECs.

### ECs express ligands for integrins LFA-1 and VLA-4

Integrins LFA-1 (αLβ2) and VLA-4 (α4β1) and are known to engage endothelial-expressed cell adhesion molecules, ICAM-1 and VCAM-1, respectively, during cellular transmigration into inflamed tissues. To confirm that these ligands were expressed on the primary ECs in this coculture model, HUVECs and LECs were analyzed by flow cytometry for surface ICAM-1 and VCAM-1, as well as MAdCAM-1, the ligand for integrin α4β7, under steady-state and inflammatory (TNFα-treated) conditions. HLA DR (MHC-II) was also measured to address whether ECs might be able to activate rCD4 through TCR engagement, as was previously observed using an allogeneic stimulation model.^18,19^ IFNγ treatment of ECs was included as a positive control for upregulation of HLA DR expression.

**FIG. 4.**
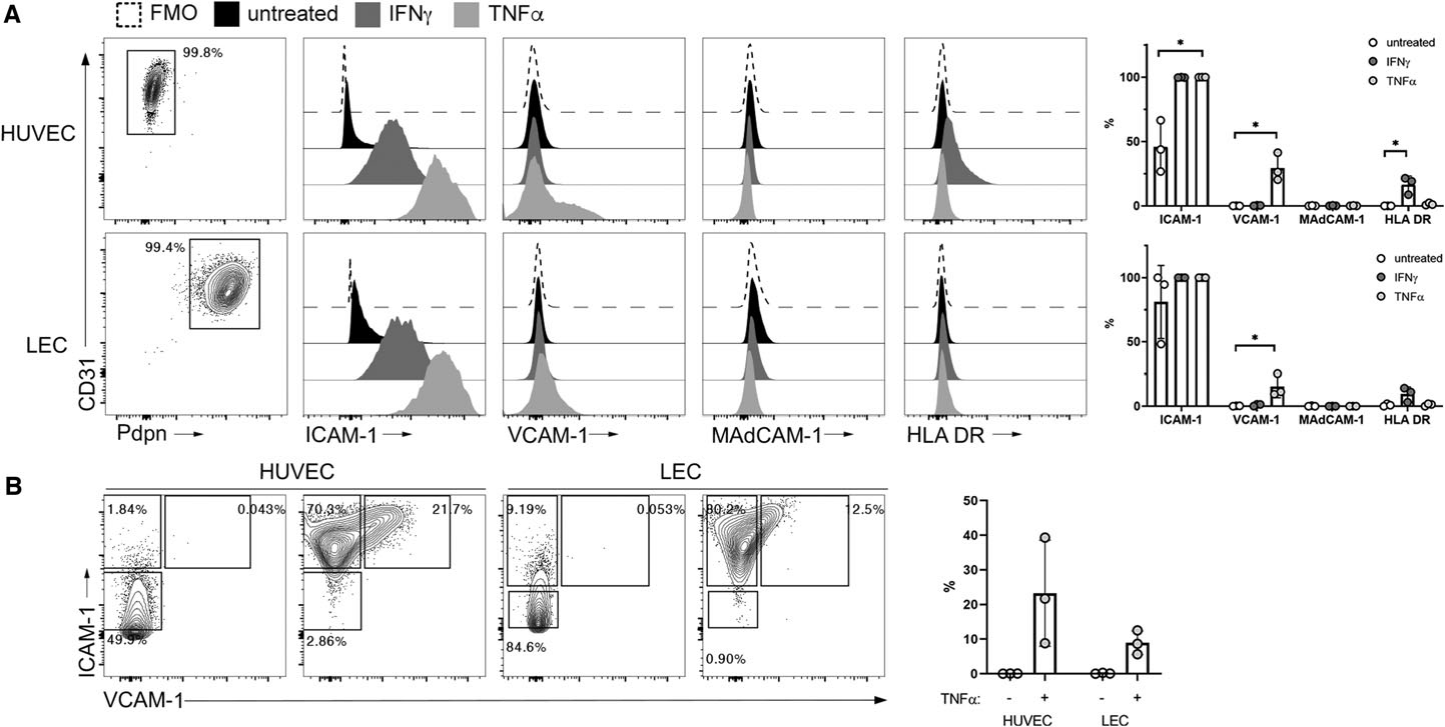
Cell adhesion molecules are expressed by ECs and upregulated under inflammatory conditions. **(A)** Representative flow cytometry data and quantification of expression of ICAM-1, VCAM-1, MAdCAM-1, and HLA DR on untreated (*black* histograms), IFNγ-treated (*dark gray*), or TNFα-treated (*light gray*) HUVECs (*top*) and LECs (*bottom*). Cells were first gated on live CD45^−^ CD31^+^ cells then were analyzed for the different phenotypic markers using FMO controls (*dashed line*). **(B)** Flow cytometry analysis of ICAM-1 and VCAM-1 coexpression on untreated or TNFα-treated ECs. Graphs show quantification of flow cytometry data from three independent experiments. Statistics assessed by Kruskal–Wallis **(A)** test and Dunn's post-test (*p < .05 in post-test comparisons) or two-way ANOVA **(B)**. ANOVA, analysis of variance; FMO, fluorescence minus one; LECs, lymphatic microvascular endothelial cells.

ICAM-1 was moderately expressed by both HUVEC and LEC under steady-state conditions and was upregulated following TNFα stimulation (Fig. 4A). VCAM-1 was similarly upregulated by both HUVEC and LEC following treatment with TNFα and the VCAM-1^+^ population coexpressed high levels of ICAM-1 (Fig. 4B). As expected, HUVECs and LECs were negative for both MAdCAM-1 and HLA DR at both steady state and following treatment with TNFα, whereas HLA DR was upregulated by IFNγ stimulation on HUVECs, but not LECs (Fig. 4A).

### EC-mediated enhancement of HIV infection of rCD4 cells is dependent on integrins

To determine whether engagement of integrins by ECs was necessary for enhancement of rCD4 infection, we used integrin-specific blocking antibodies to interfere with this interaction. We elected to use integrin α-chain-specific antibodies with the aim of preserving the ability to detect the integrin β chains by flow cytometry following blocking. Antibody clones TS1/22 (anti-LFA1 αL chain) and 2B4 (anti-VLA-4 α4 chain) were selected based on previous reports of blocking capability.^46,47^ Blocking of αL did not affect detection of LFA-1 (β2 chain), but unfortunately, blocking of α4 did interfere with staining of integrins VLA-4 (β1 chain) and α4β7 (β7 chain), precluding further detection of integrin expression following blocking (Supplementary Fig. S2).

Incubation of rCD4 cells with anti-LFA-1 (TS1/22) and anti-VLA-4 (2B4) inhibited adhesion to both TNFα-treated HUVECs and recombinant ICAM-1 or VCAM-1-coated plates in a fluorescence-based static adhesion assay, compared with isotype control (mouse IgG1κ, clone 11711, Supplementary Fig. S3).

**FIG. 5.**
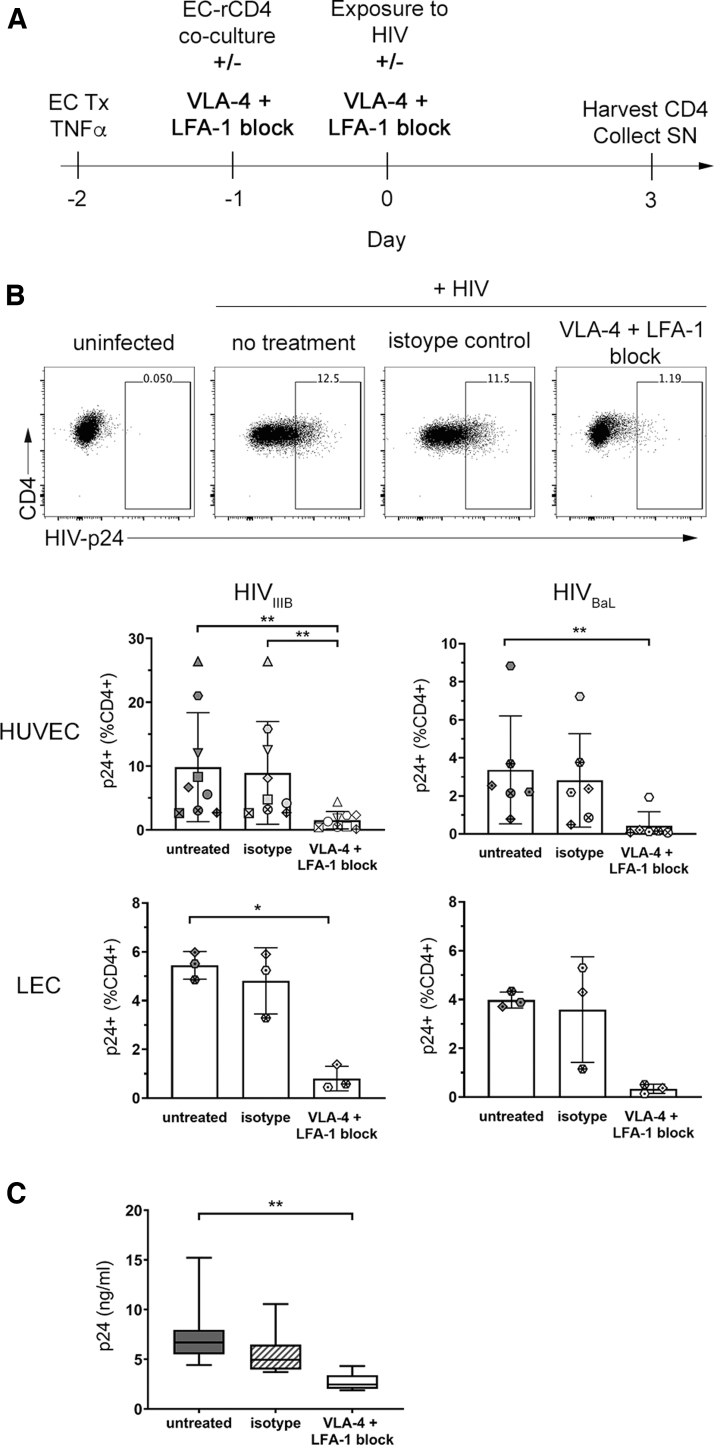
Integrin blockade prevents EC-mediated enhancement of rCD4 infection by HIV. **(A)** Schematic timeline of EC-rCD4 coculture infection assay with integrin blockade. **(B)** Representative flow cytometry data showing intracellular detection of HIV-p24 protein in rCD4 cells cocultured with HUVECs in the absence of treatment or after treatment with anti-LFA-1 (αL chain, clone TS1/22) and anti-VLA-4 (α4 chain, clone 2B4) or isotype control antibody (clone 11711). Gating for singlets, lymphocytes, live cells, CD45^+^, and CD4^+^ was performed before analyzing HIV-p24. Graphs show proportions of rCD4 cells that were HIV-p24^+^ following HIV_IIIB_ or HIV_BaL_ infection in the presence of indicated ECs and treatments. Data points indicate different CD4^+^ T cell donors and each donor is indicated by a different symbol (*n* = 3–9). **(C)** Quantification of HIV-p24 protein in supernatants from HUVEC cocultures infected with HIV_IIIB_. Statistics assessed by Friedman test with Dunn's post-test (*p < .05, **p < .01 in post-test comparisons).

Blockade of LFA-1 and VLA-4 in the coculture infection model (Fig. 4A) resulted in 65.4%–96.2% (median 85.2%) inhibition of HIV_IIIB_ and 78.1%–96.9% (median 93.4%) inhibition of HIV_BaL_ infection of rCD4s cocultured with TNFα-treated HUVECs (*p* = .0002 and *p* = .0017, respectively, Fig. 5B). Integrin blockade similarly resulted in 76.9%–91.8% (median 88.0%) inhibition of HIV_IIIB_ infection of rCD4 cocultured with TNFα-treated LECs (*p* = .0278, Fig. 5B). Although an 88.2%–96.4% (median 90.0%) reduction in HIV_BaL_ infection of rCD4 was also observed in TNFα-treated LEC cocultures, the difference was not statistically significant, given the small sample size for the LEC cocultures (*n* = 3).

In contrast to integrin blocking antibodies, isotype control antibody had no effect on HIV infection under any conditions (Fig. 5B). Consistent with the detection of intracellular HIV-p24 in infected cells, HIV-p24 protein levels were significantly reduced in supernatants collected from cocultures with LFA-1 and VLA-4 blockade in HUVEC-TNFα cocultures (*p* = .0007; Fig. 5C). Collectively, these observations implicate cell/cell interactions between ECs and rCD4 T cells through integrin and adhesion molecules as necessary for EC-mediated enhancement of productive HIV-1 infection of rCD4 T cells.

### Enhancement of HIV infection of rCD4 is partially mediated by transinfection

**FIG. 6.**
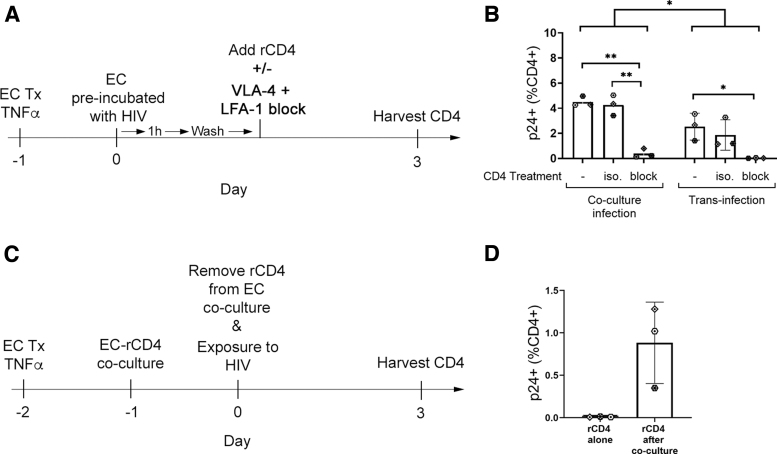
ECs promote transinfection of rCD4 cells in an integrin-dependent manner. **(A)** Schematic timeline of EC-mediated transinfection assay with integrin blockade. **(B)** Quantification of rCD4 cells that were HIV-p24^+^ following HIV_IIIB_ exposure in the established coculture assay (coculture infection) compared with rCD4 cells that were HIV-p24^+^ following coculture with TNFα-treated HUVECs preincubated with HIV_IIIB_ (transinfection; *n* = 3). Treatment with anti-LFA-1 (αL chain, clone TS1/22) and anti-VLA-4 (α4 chain, clone 2B4) or isotype control antibody (clone 11711) was performed where indicated. **(C)** Schematic timeline showing HIV infection of rCD4 following removal from coculture with ECs. **(D)** Quantification of rCD4 cells that were HIV-p24^+^ following HIV_IIIB_ exposure after removal from coculture with TNFα-treated HUVECs. Statistics assessed by two-way ANOVA with Tukey's multiple comparisons (*p < .05, **p < .01 in post-test comparisons) **(A)** or Wilcoxon test **(B)**.

Transinfection occurs when HIV virions are captured by surface proteins on cells in the microenvironment then transferred to HIV target CD4^+^ T cells. To determine whether ECs promote infection of rCD4 by transinfection, TNFα-treated HUVECs were incubated with HIV_IIIB_ for 1 h, then cells were washed to remove the viral inoculum before coculture with rCD4 (Fig. 6A).

Exposure of rCD4 to HUVECs preincubated with HIV_IIIB_ resulted in enhancement of infection, although at lower levels than was observed in the established coculture model (*p* = .049, Fig. 6B). Similar to the observations from coculture infections, blockade of LFA-1 and VLA-4 inhibited transinfection (*p* = .018, Fig. 6B), suggesting that ECs can promote transinfection of rCD4 in an integrin-dependent manner.

To determine whether transinfection was the sole mechanism promoting infection, rCD4 were cocultured with TNFα-treated HUVECs for 24 h then removed from coculture before exposure to HIV_IIIB_ (Fig. 6C). In contrast to rCD4 cultured alone, rCD4 removed from cocultures with TNFα-treated HUVECs did demonstrate susceptibility to HIV infection (Fig. 6D), but this effect was not statistically significant with the low sample size available for these experiments (*n* = 3).

### Coculture of rCD4 with ECs does not affect integrin expression but induces activation in a small proportion of cells

To determine whether ECs modulated expression of the identified HIV susceptibility markers on rCD4s, rCD4 cells were evaluated for expression of integrins VLA-4 (β1), LFA-1 (β2) and α4β7 (β7), and CCR6 on naive (CD45RA^+^) and memory (CD45RA^−^) subsets and markers of activation (CD69, Ki67) after being cultured alone or with TNFα-treated HUVECs in the absence of HIV for 4 days (equivalent to total coculture time in infection assays). Phenotypes after culture were compared with baseline phenotypes acquired immediately after rCD4 isolation (Table 2).

**Table 2.** Comparison of Phenotypes Between *Ex Vivo* Isolated rCD4 and rCD4 After Culture for 4 Days Alone or with TNFα-Treated Human Umbilical Vein Endothelial Cell

Parameter^a^	Ex vivo	4 Days culture		Friedman* p *value^b^	Dunn's pairwise comparisons^c^		
	rCD4	rCD4 alone	HUVEC-TNFα		A	B	C
CD4^+^ T cell populations							
CD69 (% CD4^+^)	0.06 (0.03–0.42)	0.05 (0.03–1.40)	3.30 (2.01–4.05)	**.0035**	ns	^*^	^*^
Ki67 (% CD4^+^)	0.09 (0.05–0.45)	0.04 (0.02–0.09)	1.15 (0.61–0.57)	**<.0001**	ns	ns	^***^
Naive CD4^+^ T cell populations							
β1^lo^ (% CD45RA^+^)	99.6 (99.4–99.8)	99.2 (98.6–99.5)	99.1 (98.5–99.7)	.064			
β1 MFI	853.0 (742.5–895.5)	735.0 (615.0–910.5)	793.0 (756.0–911.5)	.569			
β2^+^ (% CD45RA^+^)	99.9 (99.8–100.0)	99.8 (99.7–100.0)	99.7 (99.4–100)	.123			
β2 MFI	5,552 (1,778–5,858)	3,007 (1,251–3,399)	4,334 (1,122–3,399)	**.0002**	^**^	^*^	ns
β7^int^ (% CD45RA^+^)	54.3 (43.3–61.7)	65.8 (57.4–77.9)	71.0 (54.7–77.3)	**.0013**	^**^	ns	ns
β7 MFI	944.0 (452.5–1,058)	1,081 (430.0–1,483)	1,012 (493.5–1,390)	.154			
Memory CD4^+^ T cell populations							
β1^hi^ (% CD45RA^−^)	52.9 (48.6–58.3)	47.8 (41.5–70.1)	58.4 (43.4–67.2)	.814			
β1 MFI	2,021 (1,912–2,596)	2,899 (2,733–3,511)	2,766 (2,536–3,728)	**.0003**	^**^	^**^	ns
β1^lo^ (% CD45RA^−^)	38.7 (36.4–48.3)	48.9 (24.2–54.8)	36.9 (27.3–53.4)	.814			
β1 MFI	720.0 (628.5–853.0)	905.0 (842.5–949.0)	807.0 (787.5–998.0)	**.0013**	^**^	ns	ns
β2^hi^ (% CD45RA^−^)	99.8 (99.8–100.0)	99.8 (98.8–100.0)	99.9 (99.8–100)	.329			
β2 MFI	8,053 (2,981–9,328)	5,660 (2,965–6,491)	9,743 (2,234–13,046)	.278			
β7^hi^ (% CD45RA^−^)	25.6 (20.9–31.6)	29.2 (20.9–40.6)	29.9 (26.2–37.4)	**.0035**	^*^	^*^	ns
β7^hi^ MFI	3,388 (1,971–3,733)	6,892 (2,560–7,272)	5,297 (2,887–6,013)	**<.0001**	^***^	^*^	ns
CCR6 (% CD45RA^−^)	40.2 (31.2–49.2)	29.5 (23.8–38.5)	29.0 (18.8–34.2)	**.0007**	ns	^**^	ns
CCR6 MFI	2,433 (2,230–2,824)	2,193 (2,088–2,464)	2,728 (1,879–3,009)	.741			

^a^ Expression values listed as median of % positive (+) cells in indicated parent population or MFI, with interquartile range shown in parentheses.

^b^ *p*-Values calculated using nonparametric Friedman test. Parameters considered statistically significant (*p* < .05) are highlighted in bold text.

^c^ Dunn's multiple comparisons test used for pairwise comparisons in the case of Friedman ANOVA *p* < .05. A = *ex vivo* rCD4 versus rCD4 cultured alone; B = *ex vivo* rCD4 versus rCD4 cocultured with HUVEC-TNFα; C = rCD4 cultured alone versus rCD4 cocultured with HUVEC-TNFα.

^*^ *p* < .05; ^**^p < .01; ^***^p < .001.

ANOVA, analysis of variance; HUVEC, human umbilical vein endothelial cell; ns, not significant; rCD4, resting CD4^+^ T cell.

*In vitro* culture affected the phenotype of rCD4, such that naive rCD4 cells had reduced β2 expression density (MFI; *p* = .0002) and elevated proportions of β7^hi^ cells (*p* = .0013) when rCD4 were either cultured alone, or with EC, relative to freshly isolated rCD4. Similarly, memory (CD45RA^−^) rCD4 that were cultured *in vitro* had increased β1 expression density in the β1^hi^ (MFI; *p* = .0003) and β1^lo^ (MFI; *p* = .0013) subsets, increased proportion of cells that were β7^hi^, with increased antigen density of β7 on those cells (MFI; *p* < .0001), and reduced proportions of CCR6^+^ cells (*p* = .0007). However, none of these phenotypes differed between rCD4 that were cultured alone and those that were cultured in the presence of ECs (Table 2), indicating that *in vitro* culture, rather than ECs, induced the observed phenotypic changes.

In contrast, higher proportions of CD69^+^ and Ki67^+^ rCD4s were observed following HUVEC coculture, whereas rCD4 cells cultured alone remained in a resting state, with similar proportions of CD69- and Ki67-expressing cells as freshly isolated rCD4 (medians CD69^+^ 0.05%, Ki67^+^ 0.04%). However, despite the relative increases in CD69 and Ki67, the overall proportions of activated cells remained low (median CD69^+^ 3.3%, median Ki67^+^ 1.2%). Furthermore, as shown in Figure 3, CD69 cells were not depleted from the uninfected (HIV-p24^−^) subset after exposure to HIV_IIIB_, suggesting that the increase in activation did not account for the enhancement of HIV infection observed. Indeed, in matched samples, there were significantly more rCD4 cells that became infected (median 6.7%) than those that became activated (median 3.3%) when cocultured with TNFα-treated HUVECs (*p* = .008) (Supplementary Fig. S4).

Taken together, these data suggest that the EC-mediated enhancement of rCD4 infection by HIV is mediated by cellular activation.

## Discussion

Resting CD4 cells are known to be early HIV targets *in vivo*, in contrast to their relative resistance to infection *in vitro*. There is an established role for the tissue microenvironment, and ECs in particular, in enhancing HIV susceptibility of rCD4 cells.

In this study, we have demonstrated that in the presence of ECs, HIV infection of rCD4 cells occurs primarily in a subset of memory cells expressing high levels of integrins LFA-1 and VLA-4. ICAM-1 and VCAM-1, the ligands for LFA-1 and VLA-4 respectively, were expressed by TNFα-treated ECs. Blocking of these integrins prevented HIV infection in our coculture model. ECs were able to mediate transinfection in an integrin-dependent manner, but other mechanisms may also contribute to enhancement of infection. To our knowledge, this is the first demonstration of EC-mediated enhancement of HIV infection of rCD4 cells, which occurs in an integrin-dependent manner.

ICAM and LFA-1 are critically important in cell/cell spread of HIV, either between homotypic CD4^+^ T cells or through transinfection from an alternate cell type that has captured HIV virions, such as a dendritic cell.^40,41^ Transinfection is not limited to dendritic cells, and has been described for ECs^48,49^ and other nonhematopoietic cell types, including epithelial cells,^50,51^ fibroblasts,^50,52^ and lymph node fibroblastic reticular cells.^53^

In the present study, when ECs were preincubated with virus then washed before coculture with rCD4 cells, enhancement of rCD4 infection was observed, although at a lower level than what was observed in matched coculture infections. When rCD4 cells were treated with integrin blocking antibodies, infection was abrogated. These data suggest that ECs can promote infection of resting memory CD4^+^ T cells through transinfection in an integrin-dependent manner. This may occur through capture of integrin-decorated HIV virions through ICAM or VCAM proteins and subsequent transfer to target cells. LFA-1/ICAM-1 interactions may further contribute to this process through stabilization of cell/cell interactions.^41^

While transinfection partially mediated EC-mediated enhancement of HIV infection of rCD4 cells, rCD4 cells removed from EC coculture before exposure to HIV still demonstrated enhanced susceptibility to infection, although this was not statistically significant in our dataset. This effect has been previously observed,^21^ suggesting that mechanisms other than transinfection contribute to enhancement of rCD4 infection by ECs.

Adhesion molecules have been implicated in promoting HIV infection of CD4^+^ T cells in multiple ways. Both HIV_IIIB_ and HIV_BaL_ used in our study were propagated in physiologically relevant human PBMCs and can be expected to bear host proteins, including integrins and CAMs. Indeed, previous studies have demonstrated incorporation of host proteins, including integrins α4, αL, β7, β1, ICAM-1, and others into the viral envelope of virions propagated in human PBMCs.^37,54–58^ HIV virions bearing ICAM-1^57,59^ and α4β7^54^ have been shown to directly bind their cognate ligands on target cells to promote viral entry.^39,60^ As such, it is possible that virion-embedded host ICAM-1 could engage LFA-1 on target resting memory CD4^+^ T cells to facilitate infection. However, infection of control rCD4 cells cultured in the absence of ECs was not observed, demonstrating that enhanced infection of rCD4 cells occurs in an EC-dependent manner and cannot be explained by direct binding by virion-embedded CAMs.

In support of this, preferential HIV entry into α4β7 and VLA-4-expressing cervical CD4^+^ T cells was previously shown to occur independently of direct HIV binding to integrins.^24^

In addition to promoting infection of rCD4 cells through potential virion capture and transinfection, ECs directly engage integrins, leading to downstream changes that may allow the cells to support HIV replication. CD4^+^ T cell activation typically involves engagement of the TCR by antigen-complexed MHC-II molecules in combination with a costimulatory signal. Indeed, early studies using an allogeneic stimulation model implicated MHC-II expressed by IFNγ-treated ECs in enhancement of HIV infection of resting CD4^+^ T cells,^18,19^ and α_4_β_7_ has been shown to provide costimulation to TCR-activated CD4^+^ T cells when engaged by MAdCAM-1, thereby promoting productive HIV replication *in vitro*.^61^ However, ECs do not express MHC-II under steady-state conditions and require IFNγ stimulation for upregulation of this pathway.

We confirmed that the HUVECs and LECs used in our model did not express MHC-II under resting conditions or after TNFα stimulation but could upregulate MHC-II after IFNγ treatment. In line with this, minimal cellular activation of CD4^+^ T cells was observed after coculture with HUVECs in the absence of HIV, as indicated by expression of the acute activation marker CD69 and the proliferation marker Ki67. However, following HIV infection, approximately one third of infected cells expressed the activation marker CD69, suggesting upregulation due to viral infection. In contrast, Ki67 expression among infected cells remained low, indicating that despite induction of CD69, infected cells were not actively proliferating.

Coexpression analysis revealed that the integrin-expressing cells identified as the primary HIV target cell did not consistently express CD69, suggesting that engagement of integrins on rCD4 target cells by CAMs on ECs does not result in cellular activation. We observed that rCD4 expressing high levels of LFA-1 and VLA-4 were depleted from the uninfected population in cocultures exposed to HIV, suggesting preferential infection of those cells. However, our data do not exclude the possibility that integrin expression was upregulated following infection, contributing to the overall enrichment of these markers among infected cells. In contrast, CD69-expressing cells were maintained in the uninfected population, suggesting they were not preferentially targeted for infection.

Collectively, these data are consistent with the results of previous studies that showed minimal contribution of cellular activation to enhancement of resting CD4^+^ T cells by ECs.^20^ Nevertheless, we cannot exclude the possibility that this low-level cellular activation may promote infection of a small subset of target CD4^+^ T cells. As this analysis was limited by the use of only two dynamic activation markers, CD69 and Ki67, at a single cross-sectional time point, a more comprehensive examination of rCD4 activation by ECs and the relative contribution of cellular activation to the enhancement of HIV infection is warranted.

Other downstream consequences of integrin engagement may also promote establishment of productive infection. For instance, integrin signaling is a tightly controlled and dynamic process that is largely dependent on remodeling of the actin cytoskeleton. Several stages of HIV replication are similarly dependent on actin remodeling.^62^ Cytoskeletal mobilization was previously implicated in enhanced entry of ICAM-bearing viruses into LFA-1-expressing CD4^+^ T cells.^63^ Similar intersecting pathways may play a role in the integrin-dependent enhancement of HIV infection in resting CD4^+^ T cells by ECs.

In addition to integrins, the trafficking molecule CCR6 was enriched among infected CD4^+^ T cells in our EC coculture model. CCR6 is expressed on Th17, Th1Th17, and Th22 CD4^+^ T cell subsets and binds to CCL20 to mediate cellular migration of cells to barrier tissues such as the gut and female reproductive tract.

Previous reports identified CCR6^+^ CD4^+^ T cells as preferentially infected by HIV *in vitro*^64,65^ and *in vivo*^66,67^ and constitute the initial founder population of infected cells in the female reproductive tract of SIV-infected nonhuman primates.^68^ These cells are enriched for HIV-dependency factors^69^ and contribute to persistence of the HIV reservoir.^70^ Ligand binding to chemokine receptors such as CCR6 leads to integrin activation and enables resting cells to migrate into inflamed tissues in a manner dependent on cytoskeletal changes.^71^ These pathways are important for HIV infection, and CCL20 signaling through CCR6 was previously shown to overcome barriers to HIV infection in resting CD4^+^ T cell and promote HIV latency.^72^ CCL20 is produced by a variety of cell types in mucosal tissues, including ECs, epithelial cells, and various immune cell subsets.^73^

Future work will investigate chemokine production, in particular CCL20, by ECs in our coculture model and downstream effects on integrin activation and CAM engagement by resting memory CD4^+^ T cells.

The potential mechanisms described in this study are not necessarily mutually exclusive. It is possible that ECs contribute to infection of resting memory CD4^+^ T cells through transinfection and through direct effects on target cells following integrin engagement. Integrin blockade may also prevent engagement of additional interactions between ECs and resting CD4^+^ T cells that are dependent on cell adhesion. There may be additional integrin-dependent or -independent mechanisms contributing to the productive infection of resting CD4^+^ T cells in the presence of ECs. Consistent with this, there was a proportion of infected cells that were not characterized by high levels of integrins or activation markers.

The observed enhancement of infection resulting from the interaction between ECs and resting memory CD4^+^ T cells has clear relevance for HIV acquisition and pathogenesis. Resting memory CD4^+^ T cells are the primary infection targets *in vivo*, and constitute a key cellular reservoir for HIV latency. Inflammation, which promotes cell/cell interactions between ECs and memory T cells through upregulation of adhesion molecules, is known to increase susceptibility to HIV acquisition. The demonstration that these same cell trafficking molecules enhance the HIV susceptibility of resting CD4^+^ T cells, which otherwise demonstrate low susceptibility to HIV infection, underscores the physiological relevance of these findings for HIV acquisition and opportunities for intervention.

## Supplementary Material

Supplemental data

Supplemental data

Supplemental data

Supplemental data

Supplemental data

Supplemental data
