# Supplementary material for: Endothelial Cells Promote Productive HIV Infection of Resting CD4+ T Cells by an Integrin-Mediated Cell Adhesion-Dependent Mechanism
Source: AIDS Res Hum Retroviruses. 2022 Feb 4;38(2):111–26. doi: 10.1089/aid.2021.0034 (PMC8861939; doi:10.1089/aid.2021.0034)
Supplement: Supplemental data [file Supp_TableS1.docx]

| Supplementary Table 1. Flow cytometry panels | | | |
| --- | --- | --- | --- |
| Marker | Clone | Fluorochrome | Source |
| Panel: rCD4 activation and Purity | | | |
| CD45 | HI30 | BUV395 | BD |
| CD4 | RPA-T4 | BV605 | Biolegend |
| CD8 | RPA-T8 | BV650 | Biolegend |
| CD3 | UCHT1 | PECF594 | BD |
| HLA DR | LN3 | SuperBright436 | eBioscience |
| CD25 | 2A3 | BB515 | BD |
| CD69 | FN50 | APCR700 | BD |
| Viability | -- | Live/Dead Aqua | Invitrogen |
| Panel: CD4 T cell Infection, Activation, Memory and Trafficking | | | |
| CD45 | HI30 | BUV395 | BD |
| CD4 | RPA-T4 | BB515 | BD |
| CD45RA | HI100 | PerCPCy5.5 | Biolegend |
| CCR7 | 3D12 | PECy7 | BD |
| CCR6 | 11A9 | BV786 | BD |
| Integrin β1 | MAR4 | BV711 | BD |
| Integrin β2 | 6.7 | APC | BD |
| Integrin β7 | FIB504 | BV421 | BD |
| CD69 | FN50 | APCR700 | BD |
| Ki67 | Ki-67 | BV605 | Biolegend |
| HIV-p24 | KC57 | RD1 | Beckman Coulter |
| Viability | -- | Live/Dead Aqua | Invitrogen |
| Panel: Endothelial Cells | | | |
| Podoplanin | NC-08 | AlexaFluor 488 | Biolegend |
| CD31 | WM59 | PerCPCy5.5 | Biolegend |
| ICAM-1 | HA58 | BV786 | BD |
| VCAM-1 | 51-10C9 | BUV737 | BD |
| MAdCAM-1 | 683715 | APC | R&D Systems |
| CD62E | 68-5H11 | BV650 | BD |
| HLA DR | L243 | BV421 | Biolegend |
| Viability | -- | Live/Dead Aqua | Invitrogen |
