# Supplementary material for: Endothelial Cells Promote Productive HIV Infection of Resting CD4+ T Cells by an Integrin-Mediated Cell Adhesion-Dependent Mechanism
Source: AIDS Res Hum Retroviruses. 2022 Feb 4;38(2):111–26. doi: 10.1089/aid.2021.0034 (PMC8861939; doi:10.1089/aid.2021.0034)
Supplement: Supplemental data [file Supp_TableS2.docx]

| Supplementary Table 2. Comparison of phenotypes between *ex vivo* PBMC and isolated resting CD4+ T cells | | | |
| --- | --- | --- | --- |
| Parameter ^a^ | PBMC | rCD4 | p-value ^b^ |
| CD4+ T cell populations | | | |
| CD69 (%CD4+) | 0.37 (0.17-0.80) | 0.06 (0.03-0.42) | **0.020** |
| Ki67 (%CD4+) | 0.44 (0.18-0.93) | 0.086 (0.05-0.45) | **0.002** |
| CD45RA (%CD4+) | 53.9 (45.0-59.5) | 65.5 (59.1-75.7) | **0.004** |
| Naïve CD4+ T cell populations | | | |
| β1^lo^ (%CD45RA+) | 99.4 (99.3-99.5) | 99.6 (99.4-99.8) | 0.094 |
| β1 MFI | 794.0 (725.5-742.5) | 853.0 (742.5-895.5) | 0.844 |
| β2+ (%CD45RA+) | 99.7 (99.4-99.9) | 99.9 (99.8-100.0) | 0.125 |
| β2 MFI | 4681 (1412-6807) | 5552 (1778-5858) | 0.098 |
| β7^int^ (%CD45RA+) | 50.2 (38.7-61.4) | 54.3 (43.3-61.7) | **0.043** |
| β7 MFI | 933.0 (437.0-1058) | 944.0 (452.5-1058) | 0.570 |
| CCR7 (%CD45RA+) | 97.7 (94.7-99.1) | 99.3 (98.8-99.9) | **0.004** |
| Memory CD4+ T cell populations | | | |
| β1^hi^ (%CD45RA-) | 38.8 (35.1-48.1) | 52.9 (48.6-58.3) | **0.027** |
| β1 MFI | 2068 (1874-2396) | 2021 (1912-2596) | 0.250 |
| β1^lo^ (%CD45RA-) | 44.4 (38.3-48.7) | 38.7 (36.4-48.3) | 0.551 |
| β1 MFI | 742.0 (652.5-812) | 720.0 (628.5-853.0) | 0.320 |
| β2^hi^ (%CD45RA-) | 99.8 (99.3-100.0) | 99.8 (99.8-100.0) | 0.094 |
| β2 MFI | 7749 (2526-9559) | 8053 (2981-9328) | 0.301 |
| β7^hi^ (%CD45RA-) | 18.7 (14.9-27.1) | 25.6 (20.9-31.6) | **0.004** |
| β7^hi^ MFI | 3450 (1762-3771) | 3388 (1971-3733) | 0.129 |
| CCR6 (%CD45RA-) | 30.5 (15.3-38.4) | 40.2 (31.2-49.2) | **0.039** |
| CCR6 MFI | 1884 (1442-2640) | 2433 (2230-2824) | **0.012** |
| CCR7 (%CD45RA-) | 74.3 (69.7-76.6) | 87.7 (84.8-91.7) | **0.004** |
| ^a^ expression values listed as median of % positive (+) cells in indicated parent population or median fluorescence intensity (MFI), with interquartile range shown in parentheses  ^b^ p-values calculated using non-parametric Wilcoxon matched pairs test. Parameters considered statistically significant (p<0.05) are highlighted in bold text | | | |
