# Supplementary material for: Endothelial Cells Promote Productive HIV Infection of Resting CD4+ T Cells by an Integrin-Mediated Cell Adhesion-Dependent Mechanism
Source: AIDS Res Hum Retroviruses. 2022 Feb 4;38(2):111–26. doi: 10.1089/aid.2021.0034 (PMC8861939; doi:10.1089/aid.2021.0034)
Supplement: Supplemental data [file Supp_FigS1.docx]

**
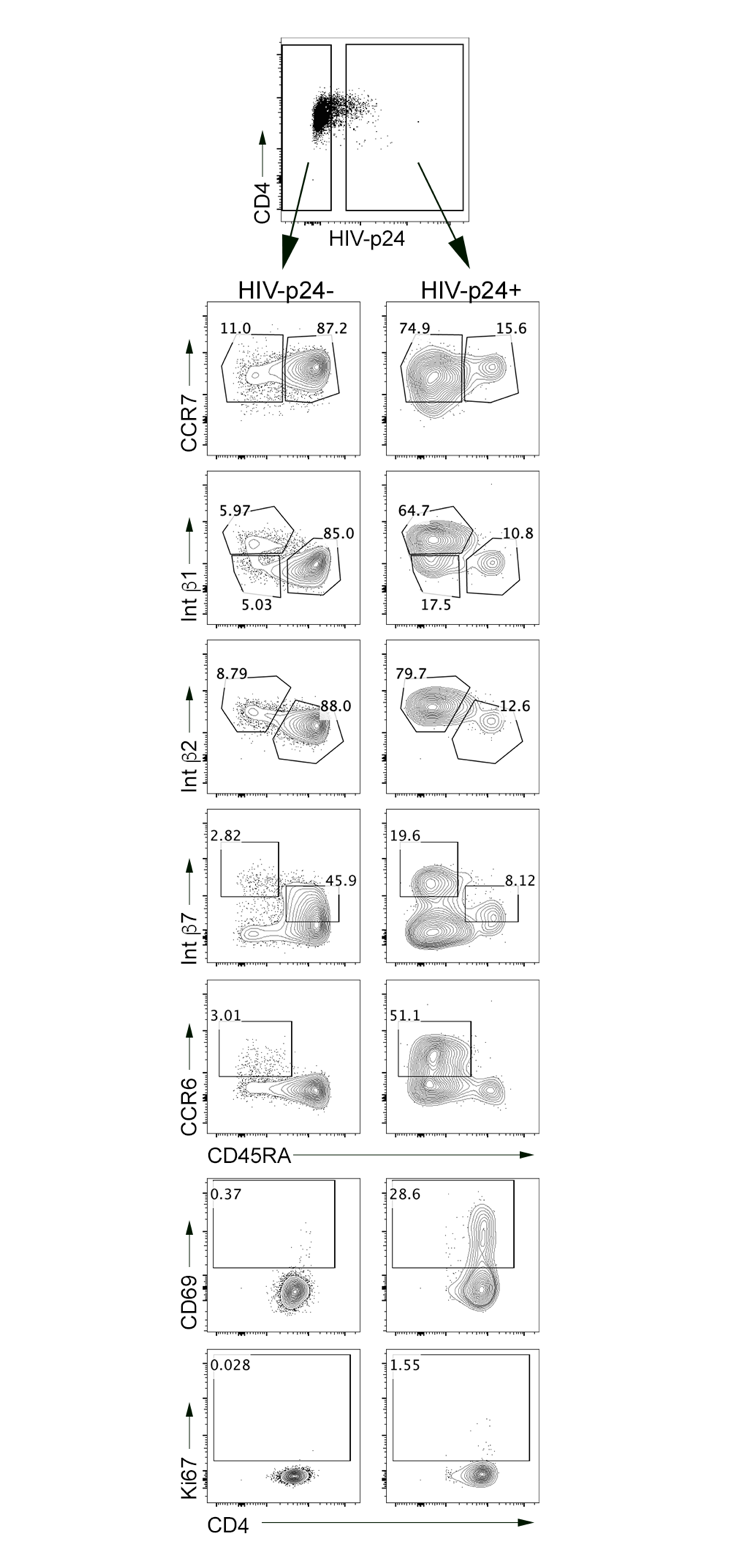
**

**Supplementary Fig. 1. Phenotypic comparison of HIV-infected and uninfected rCD4.** Representative flow cytometry showing characterization of HIV-uninfected (HIV-p24-) and HIV-infected (HIV-p24+) rCD4 cells from co-cultures with TNFα-treated ECs assessed for markers of activation (CD69, Ki67), memory (CD45RA, CCR7), integrins VLA-4 (β1), LFA-1 (β2) and α4β7 (β7) and CCR6. Gating for singlets, lymphocytes, live cells, CD45+ and CD4+ was performed before analyzing HIV-p24. Quantification and statistical comparisons are shown in Table 1.
