# Supplementary material for: Endothelial Cells Promote Productive HIV Infection of Resting CD4+ T Cells by an Integrin-Mediated Cell Adhesion-Dependent Mechanism
Source: AIDS Res Hum Retroviruses. 2022 Feb 4;38(2):111–26. doi: 10.1089/aid.2021.0034 (PMC8861939; doi:10.1089/aid.2021.0034)
Supplement: Supplemental data [file Supp_FigS2.docx]

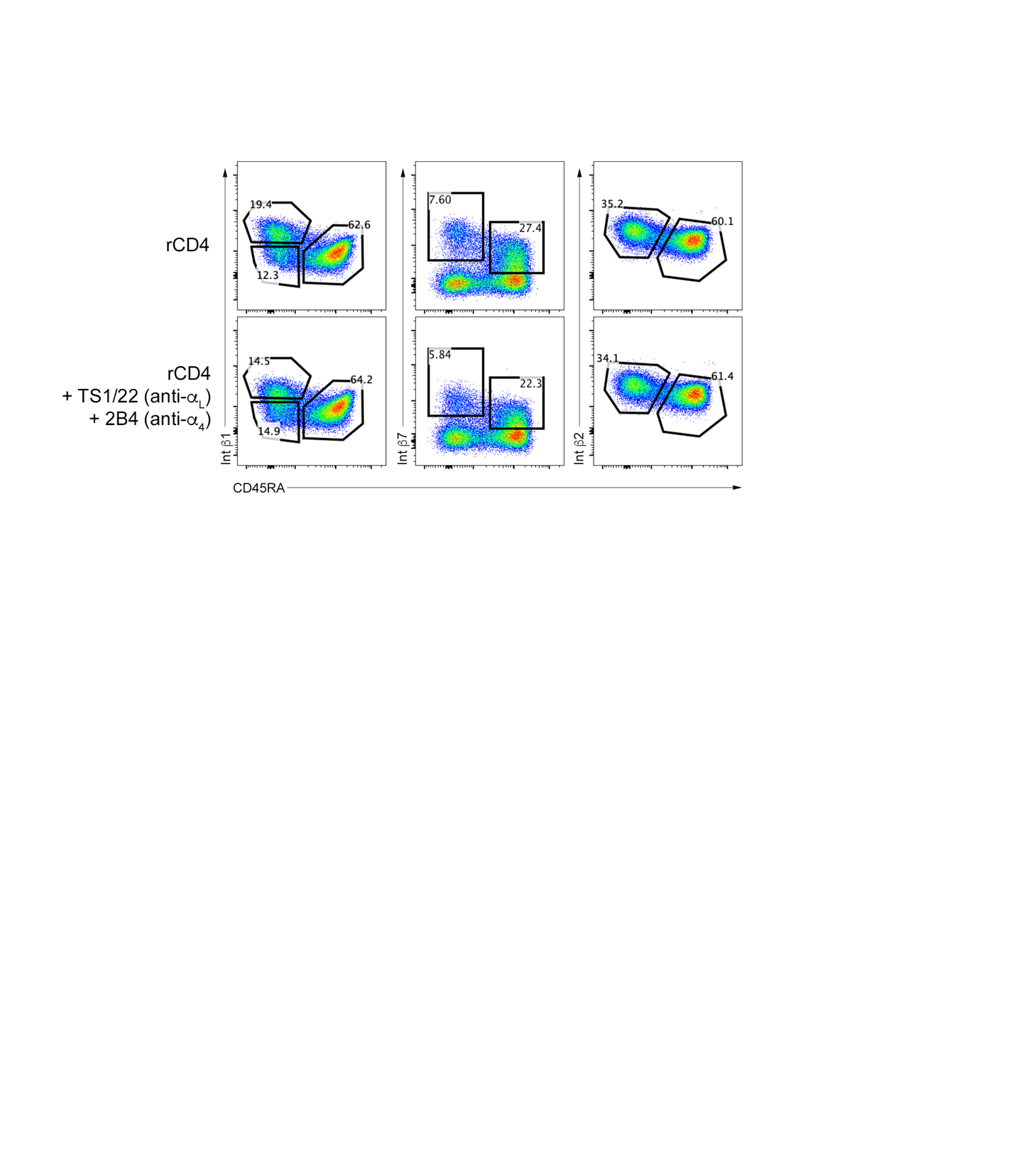


**Supplementary Fig. 2.** **Anti-α4 antibody blocks detection of β1 and β7 integrin chains**. rCD4 cells were cultured alone or in the presence of anti-αL (clone TS1/22) and anti-α4 (clone 2B4) for 30 minutes before staining for integrin β chains β1, β2 and β7. Blocking interfered with detection of integrin β1 (β chain of VLA-4) and β7 (β chain of α4β7) but not β2 (β chain of LFA-1).
