# Supplementary material for: Endothelial Cells Promote Productive HIV Infection of Resting CD4+ T Cells by an Integrin-Mediated Cell Adhesion-Dependent Mechanism
Source: AIDS Res Hum Retroviruses. 2022 Feb 4;38(2):111–26. doi: 10.1089/aid.2021.0034 (PMC8861939; doi:10.1089/aid.2021.0034)
Supplement: Supplemental data [file Supp_FigS3.docx]

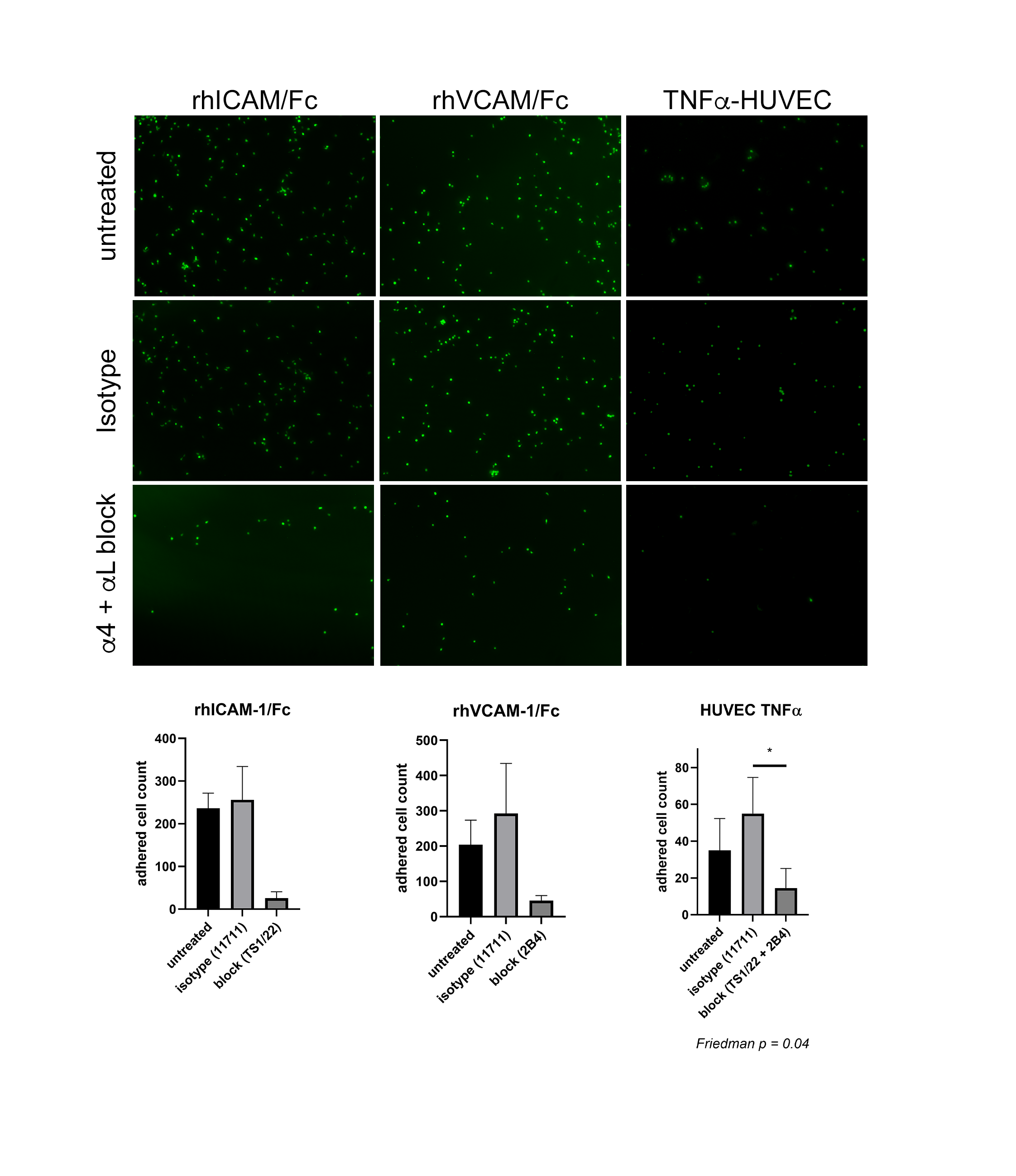


**Supplementary Fig. 3. Integrin-specific blocking antibodies prevent cell adhesion.** CD4+ T cells were labelled with Cell Tracker Green then treated with antibodies specific for LFA-1 (αL, clone TS1/22), VLA-4 (α4, clone 2B4), an isotype control (IgG1 clone 11711) or left untreated. Cells were then allowed to adhere to either rhICAM-1/Fc, rhVCAM-1/Fc or a monolayer of TNFα-treated HUVECs then non-adhered cells were removed and adhered cells were visualized using fluorescence microscopy.
