# Supplementary material for: Endothelial Cells Promote Productive HIV Infection of Resting CD4+ T Cells by an Integrin-Mediated Cell Adhesion-Dependent Mechanism
Source: AIDS Res Hum Retroviruses. 2022 Feb 4;38(2):111–26. doi: 10.1089/aid.2021.0034 (PMC8861939; doi:10.1089/aid.2021.0034)
Supplement: Supplemental data [file Supp_FigS4.docx]

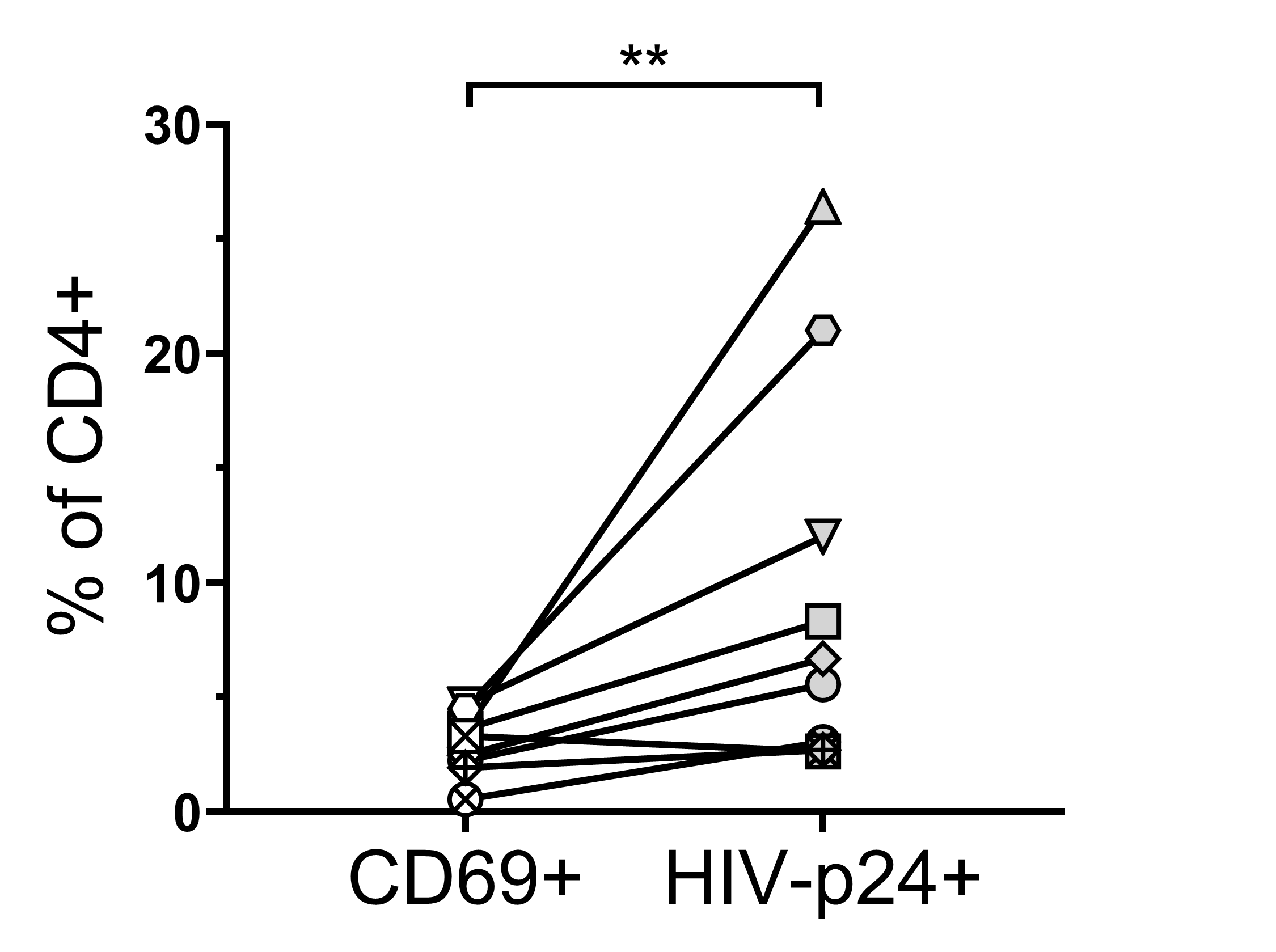


**Supplementary Fig. 4.** **Greater proportions of rCD4 become infected with HIV than those that express CD69 when co-cultured with EC.** Comparison of proportions of CD4+ cells that became activated after co-culture with TNFα-treated ECs (CD69+) and cells that became infected after co-culture with TNFα-treated ECs and exposure to HIV_IIIB_ (HIV-p24+).
